# Molecular and morphological studies on *Contracaecum rudolphii* A and *C. rudolphii* B in great cormorants (*Phalacrocorax carbo sinensis*) from Italy and Israel

**DOI:** 10.1017/S0031182023000902

**Published:** 2023-09

**Authors:** Monica Caffara, Perla Tedesco, Nadav Davidovich, Silva Rubini, Valentina Luci, Alessia Cantori, Patrycja Anna Glogowski, Maria Letizia Fioravanti, Andrea Gustinelli

**Affiliations:** 1 Department of Veterinary Medical Sciences (DIMEVET), Alma Mater Studiorum University of Bologna, Bologna, Italy; 2 Israeli Veterinary Services, Bet Dagan, Israel; 3 Experimental Zooprophylactic Institute of Lombardy and Emilia Romagna, Brescia, Italy

**Keywords:** *Contracaecum rudolphii* A, *C*. *rudolphii* B, Israel, Italy, *Phalacrocorax carbo sinensis*, taxonomy

## Abstract

The distribution of parasites is shaped by a variety of factors, among which are the migratory movements of their hosts. Israel has a unique position to migratory routes of several bird species leaving Europe to winter in Africa, however, detailed studies on the parasite fauna of birds from this area are scarce. Our study investigates occurrence and distribution of sibling species among *Contracaecum rudolphii* complex in *Phalacrocorax carbo sinensis* from Italy and Israel, to acquire further information on the geographical range of these species to gain deeper knowledge on the ecology of these parasites and their bird host. A total of 2383 *Contracaecum* were collected from the gastric mucosa of 28 great cormorants (18 from Israel and 10 from Italy). A subsample was processed for morphological analyses in light and scanning electron microscopy (SEM), and for molecular analyses through amplification and sequencing of the ITS rDNA and the *cox*2 mtDNA, and through PCR-RFLP. All the 683 *Contracaecum* subjected to molecular identification belonged to *C. rudolphii s.l.*, (300 *C. rudolphii* A and 383 *C. rudolphii* B). SEM micrographs provided, for the first time, details of taxonomic structures in male specimens from both sibling species, and the first SEM characterization of *C. rudolphii* B. This work presents the first data on the occurrence of sibling species of *C. rudolphii* in Israel and provides additional information on the distribution of *C. rudolphii* A and B in Italy, confirming the high prevalence and intensity of infection observed in *Ph. carbo sinensis* from other Italian areas.

## Graphical abstract

**Figure:**
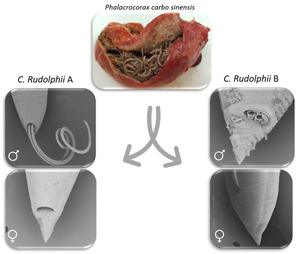

## Introduction

Members of the species complex *Contracaecum rudolphii* parasitize mainly cormorants (family Phalacrocoracidae) worldwide, with 6 sibling species displaying characteristic geographic distribution and host preference. Particularly, *C. rudolphii* A has been described in the great cormorant *Phalacrocorax carbo sinensis* (Mattiucci *et al*., 2002, 2020; Amor *et al*., 2020; Carmeno *et al*., 2022; Cammilleri *et al*., 2023), in the European shag *Ph. aristotelis aristotelis* (Abollo *et al*., 2001) and *Ph. aristotelis desmarestii* (Roca-Geronès *et al*., 2023) from Europe; *C. rudolphii* B parasitizes *Ph. carbo sinensis* (Mattiucci *et al*., 2002, 2020; Amor *et al*., 2020; Carmeno *et al*., 2022; Cammilleri *et al*., 2023) from Europe; *C. rudolphii* C is reported in the double-crested cormorant *Ph. auritus* from the USA (D'Amelio *et al*., 2007), while *C. rudolphii* D and *C. rudolphii* E are reported in *Ph. carbo* and *Ph. varius* from Australia (Shamsi *et al*., 2009). Moreover, *C. rudolphii* F is reported in the brown pelican *Pelecanus occidentalis* (family Pelecanidae) from the Gulf of Mexico (D'Amelio *et al*., 2012).

The distribution of these sibling species is shaped by the feeding ecology and migratory movements of their definitive hosts. Cormorants are piscivorous birds, feeding on a wide variety of marine, brackish and freshwater fish. The continuous ingestion of fish paratenic hosts results, in many occasions, in massive infections in cormorants, which are sometimes associated with severe gastric lesions (Rokicki *et al*., 2011). In European cormorants, the sibling species *C. rudolphii* A and B are reported, with *C. rudolphii* sp. A having a life cycle more adapted to brackish and marine ecosystems, while *C. rudolphii* B occurring mainly in freshwater ecosystems (Mattiucci *et al*., 2020; Roca-Geronès *et al*., 2023). In central and eastern Europe, both species are found, often in mixed infections, in *Ph. carbo sinensis* (Mattiucci *et al*., 2002), which are thought to feed in different environments during their migration (Frederiksen *et al*., 2018). The great cormorant *Ph. carbo sinensis* is a cosmopolitan species widely distributed in all continents (Battisti *et al*., 2008; Davidovich *et al*., 2023a) and its diet is essentially represented by fish.

In Italy, larval stages of *C. rudolphii s.l.* are reported from a number of fish species; particularly *C. rudolphii* A is found in fish from brackish and marine ecosystems, including European seabass *Dicentrarchus labrax* (Paggi *et al*., 1998; Culurgioni *et al*., 2014; Guardone *et al*., 2020; Mattiucci *et al*., 2020), gilthead sea bream *Sparus aurata* (Guardone *et al*., 2020), common sole *Solea solea* and, gobies *Gobius niger* and *G. paganellus* (Culurgioni *et al*., 2014); several studies also document its occurrence in European eel *Anguilla anguilla* from brackish waters and coastal lagoons (Paggi *et al*., 1998; Culurgioni *et al*., 2014; Dezfuli *et al*., 2016; Mattiucci *et al*., 2020). Conversely, *C. rudolphii* B has been reported in fish from freshwater environments, including chub *Squalius cephalus*, barbel *Barbus barbus*, goldfish *Carassius carassius* and big-scale sand smelt *Atherina boyeri* sampled from river and lake systems in central Italy (Mattiucci *et al*., 2020), and in common bream *Abramis brama* and in European carp *Cyprinus carpio* from other parts of Europe (Molnár *et al*., 2019). Interestingly, the absence of *C. rudolphii* B in fish sampled in brackish water was recently reported (Mattiucci *et al*., 2020), which strengthens the hypothesis that this sibling species has a life cycle adapted to freshwater ecosystems.

With respect to adult stages, parasitological data on the occurrence of sibling species of *C. rudolphii* in great cormorants are available from different areas, particularly from brackish and freshwater ecosystems of northeastern and central Italy (Li *et al*., 2005; Mattiucci *et al*., 2020), from coastal brackish water ponds in Sardinia (Amor *et al*., 2020), from freshwater ecosystems of the pre-Alpine area (Carmeno *et al*., 2022) and from coastlines and the waterways of different regions in southern Italy (Cammilleri *et al*., 2023).

Geographically, Israel has a unique position with respect to migratory routes of many bird species that winter in Africa, with tens of thousands of piscivorous birds also staying and over-wintering in Israel (Nemtzov, 2002). The only *Contracaecum* species described so far in Israel in birds are *C. micropapillatum*, *C quadripapillatum, C. gibsoni* and, *C. multipapillatum* E (Caffara *et al*., 2023); larval stages of *C. quadripapillatum* and *C. multipapillatum* E have also been described in fish (Davidovich *et al*., 2022, 2023b), while *C. multipapillatum s.l.* has been reported by Smirnov *et al*. (2021). To the author's knowledge, no information on the distribution of sibling species of *C. rudolphii* in piscivorous birds or other intermediate/paratenic hosts from Israel are available so far.

This study aimed to investigate the occurrence and distribution patterns of *C. rudolphii* A and B in *Ph. carbo sinensis* collected in Italy and Israel, to acquire further information about the geographical range of these sibling species and to gain deeper knowledge on the ecology of these parasites and their bird host.

## Materials and methods

### Contracaecum sampling

**Figure 1.**
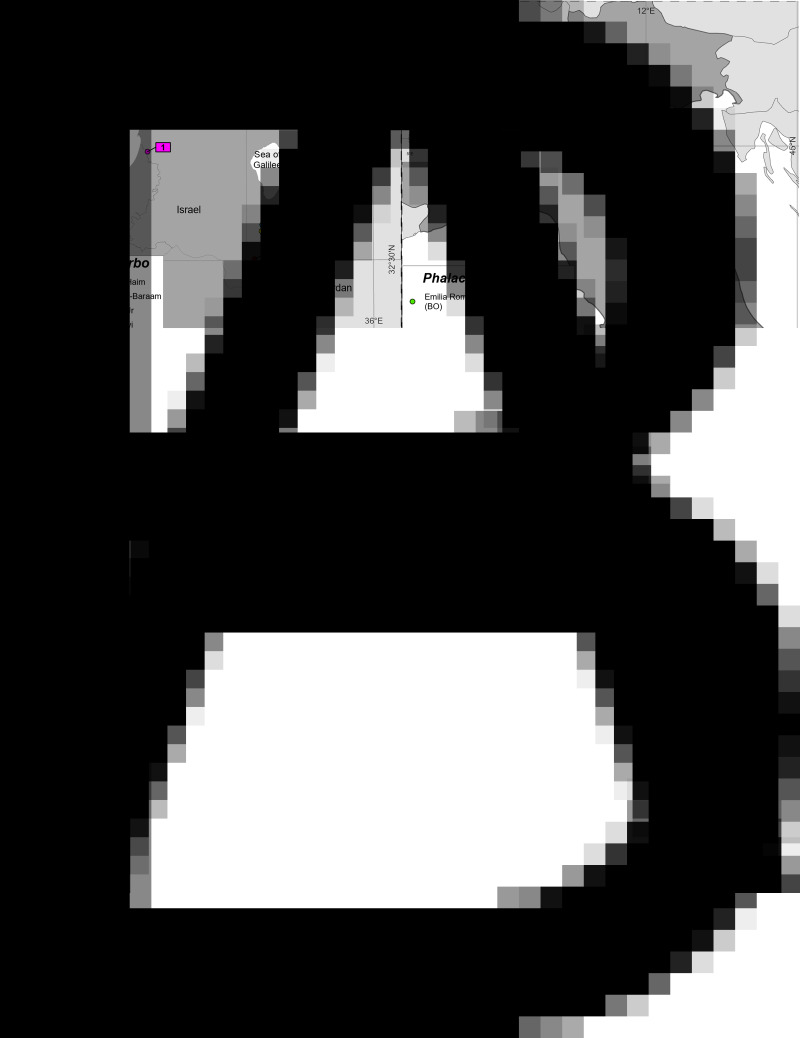
Maps of Israel (A) and Italy (B) with detail of the sampling localities together with the numbers of *Phalacrocorax carbo sinensis* collected from each locality.

Two thousand three hundred eighty-three nematodes of the genus *Contracaecum* were collected from the gastric mucosa of 28 great cormorant (*P. carbo sinensis*): 18 birds were shot from 7 localities in Israel under permits of the Israel Nature and Parks Authority 2020/42659 and 2021/42855; while 10 birds were found dead from 3 localities in Italy. In Fig. 1 (Fig. 1A, B), the number of birds collected in each locality of both countries are reported.

The nematodes were washed in saline and preserved in 70% ethanol for morphological and molecular analyses. For some adults, the anterior and posterior portions were preserved in 10% neutral-buffered formalin for SEM.

### Molecular study

For molecular analysis, genomic DNA was extracted from 683 adult males by a fast DNA extraction method using Chelex®100 (Sigma-Aldrich, Darmstadt, Germany) (Caffara *et al*., 2023). The ITS rDNA was amplified with primers NC5_f (5′-GTAGGTGAACCTGCGGAAGGATCATT-3′) and NC2_r (5′-TTAGTTTCTTCCTCCGCT-3′) (Zhu *et al*., 1998) and then 10 *μ*l were digested with the restriction endonucleases *Msp*I (*C. rudolphii* cut = 700–300 bp) and then with *Nsi*I to distinguish *C. rudolphii* A (cut = ~ 850–49 bp) and B (cut = uncut) (modified from Zhu *et al*., 2007). A fragment of the *cox*2 mtDNA was also amplified with primers 211_f (5′-TTTTCTAGTTATATAGATTGRTTTYAT-3′) and 210_r (5′-CACCAACTCTTAAAATTATC-3′) of Mattiucci *et al*. (2008) following the same protocol. From 53 specimens, both the ITS rDNA and *cox*2 mtDNA were sequenced with an ABI 3730 DNA analyser (StarSEQ, Mainz, Germany) after purification by Nucleo-Spin Gel and PCR Clean-up (Mackerey-Nagel, Düren, Germany). The DNA trace files were assembled with Contig Express (VectorNTI Advance 11 software, Invitrogen, Carlsbad, CA, USA), and the consensus sequences of the ITS rDNA and *cox*2 mtDNA were compared with published data by BLAST tools (https://blast.ncbi.nlm.nih.gov/Blast.cgi). Multiple sequence alignments were performed using BioEdit 7.2.5 (Hall, 1999), p-distance and maximum-likelihood (ML) tree (GTR + G + I substitution model for ITS, bootstrap of 1000 replicates) were obtained using MEGA 7 (Kumar *et al*., 2016). The ITS1 and ITS2 rDNA sequences were concatenated (after deleting the 5.8S rDNA) and used to build a ML tree together with the sequences of *Contracaecum* spp. reported by Mattiucci *et al*. (2020) and *Ascaris suum* (MH030604) as outgroup. The *cox*2 mtDNA gene was also aligned with the sequences reported by Mattiucci *et al*. (2020), plus *Pseudoterranova ceticola* (DQ116435) and *Anisakis pegreffii* (MT912471) as outgroups. The phylogenesis was performed by the Bayesian analysis (BI) with MrBayes 3.2.7a software (Ronquist *et al*., 2012), with GTR + G model, 4 heated Markow chains runs for 200 000 generations with sampling frequency set at 500, discarding the first 25% of the samples from the cold chain. Posterior probabilities were estimated to assess support for each branch (significant support >0.90). To infer the population genetics of *C. rudolphii* A and *C. rudolphii* B, 52 sequences newly generated plus 89 sequences of the 2 siblings from Italy, Spain and 1 from Poland, retrieved from GenBank were aligned and analysed by DnaSP V6.12.03 (Rozas *et al*., 2017): number of haplotypes (hn), diversity of haplotypes (hd), private haplotype (ph), and nucleotide diversity were determined. TCS network of haplotypes was constructed by PopART (Clement *et al*., 2000).

The sequences generated in this study have ~been deposited in GenBank under accession numbers OR263194-OR263246 (ITS rDNA) and OR269666-OR269717 (*cox*2 mtDNA).

### Morphological study

For morphological study 93 males and 13 females, randomly selected, were observed under a dissection microscope to evaluate gross morphology and to record total length (TL), then by light microscope (Leica Microsystems, Wetzlar, Germany) with the aid of a digital Nikon DS-Fi1 camera and image-acquisition software (Nikon Nis-Elements D3.0). The central part of the worms, devoid of taxonomic informative features was removed for DNA extraction. Anterior and posterior portions of the parasite body were clarified in Amman's lactophenol to measure internal structures. Morphometric analysis was carried out following Yamaguti (1935), Hartwich (1964), and Baruš *et al*. (1978).

For scanning electron microscopy (SEM), anterior and posterior portions of male and female specimens of genetically identified *C. rudolphii* A and *C. rudolphii* B were dehydrated through a graded ethanol series, dried in hexamethyldisilazane, sputter-coated with gold palladium, and observed using a Phenom XL G2 Desktop SEM (Thermo Fisher Scientific, Eindhoven, The Netherlands) operating at 5 kV.

## Results

### Molecular analyses

**Figure 2.**
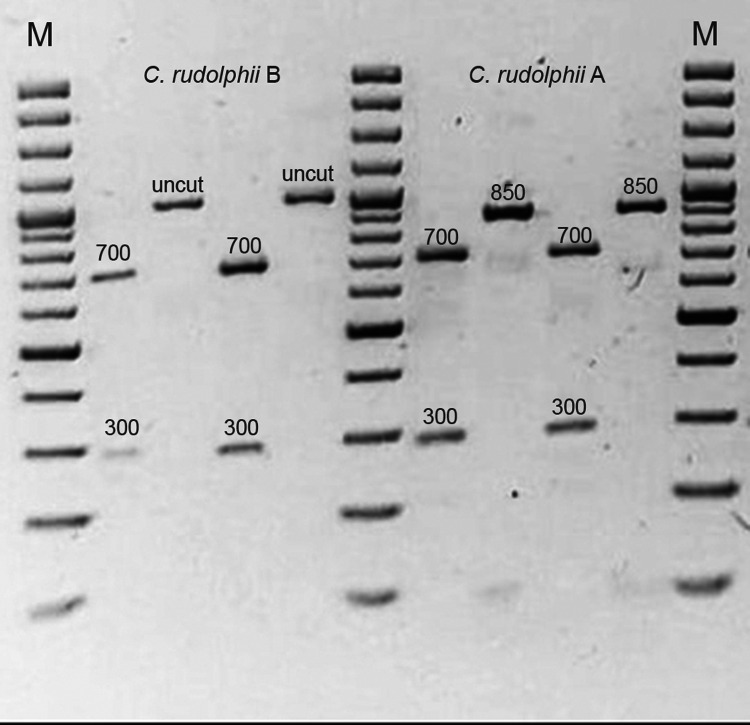
PCR-RFLP pattern of *C. rudolphii* A and *C. rudolphii* B after digestion with *Msp*I (C. rudolphii s.l.: 700-300 bp) and *Nsi*I (*C. rudolphii* A: uncut; *C. rudolphii* B: 850-49 bp).

All the 683 *Contracaecum* subjected to PCR-RFLP confirmed they belong to *C. rudolphii s.l.* and, 300 were members of the sibling species *C. rudolphii* A (*Nsi*I = 840-49 bp) while 383 were *C. rudolphii* B (*Nsi*I = uncut) (Fig. 2). Table 1 reports the distribution of the 2-sibling species together with the sampling locality for both countries under study. In most cases we detected mixed infections between the 2-sibling species, especially in cormorants from Italy.

**Table 1.** Distribution of the adults of *C. rudolphii* A and *C. rudolphii* B collected from great cormorant and the number of specimens identified by molecular methods, together with the sampling locality and years of sampling for both countries under study

Birds ID	Locality	Sampling years	Adults/mol ID	*C. rudolphii* A	*C. rudolphii* B
Israel					
PC-01	Neve Ur	Dec 2020	114/55	1	54
PC-02	Gesher	Dec 2020	70/33	10	23
PC-03			59/28	6	22
PC-04			6/6	6	0
PC-05			95/50	50	0
PC-06			38/9	6	3
PC-07	Misgav-Baraam	Nov 2021	45/10	10	0
PC-08			12/10	10	0
PC-09			64/10	0	10
PC-10	Tirat Zvi	Dec 2021	111/10	0	10
PC-16			21/16	16	0
PC-17			116/10	0	10
PC-18			82/10	0	10
PC-11	Maoz Haim	Dec 2021	49/15	1	14
PC-14			219/10	0	10
PC-12	Lohamei HaGeta'ot	Dec 2021	2/2	0	2
PC-13	Maagan Michael	Dec 2021	14/10	0	10
PC-15			49/10	0	10
**Total**			**1166**/**304**	**116**	**188**
Italy					
IT-01	Emilia Romagna (FE)	Feb 2021	51/24	11	13
IT-02			29/13	5	8
IT-03			47/17	16	1
IT-04			93/51	47	4
IT-05			18/11	7	4
IT-06			9/3	1	2
IT-07			118/60	14	46
IT-08			82/36	17	19
IT-09	Tuscany (AR)	Feb 2022	161/140	50	90
IT-10	Emilia Romagna (BO)	Dic 2021	31/24	16	8
**Total**			**639**/**379**	**184**	**195**

**Figure 3.**
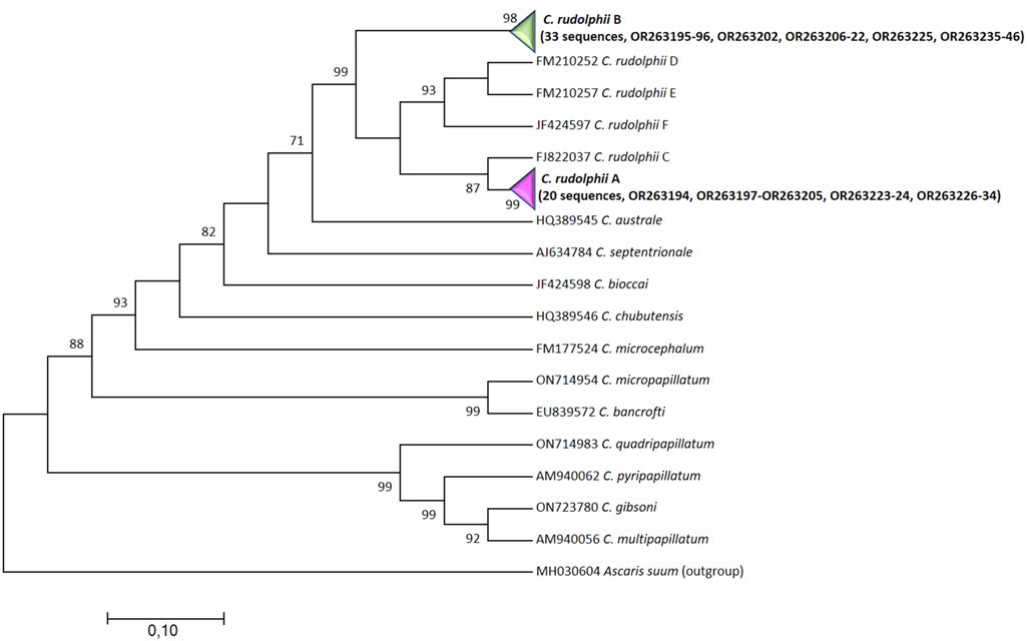
Maximum-likelihood tree based on the concatenated ITS1-ITS2 rDNA sequences showing the relationship between *C. rudolphii* A (condensed, containing 20 newly generated sequences: GenBank accession numbers OR263194, OR263197-OR263205, OR263223-24, OR263226-34, plus 2 concatenated reference sequences AJ634782 + AJ634785 of *C. rudolphii* A, Li *et al*., 2005), *C. rudolphii* B (condensed, containing 33 sequences, GB acc. n. OR263195-96, OR263202, OR263206-22, OR263225, OR263235-46, plus 2 concatenated reference sequences AJ634783 + AJ634786 of *C. rudolphii* B, Li *et al*., 2005) described in the present study (in bold) and the congeneric *Contracaecum* species. The tree is drawn to scale, with branch length measured in the number of substitutions per site.

All the sequences of the ITS rDNA of 53 specimens were of good quality and the BLAST search returned 99–100% similarity with *C. rudolphii* A (20 specimens) and *C. rudolphii* B (33 specimens). Among each sibling species the sequences were identical to each other and showed a p-distance of 0.2% between A and B-F and 0.1–0.2% between B and A-F. Interestingly the alignment of the siblings A and B newly obtained in the present study together with the other sibling species in the *C. rudolphii* complex showed the presence of an indel represented by an insertion of ‘GTTCGTGTG’ in all but not in *C. rudolphii* B. The ML tree showed a well resolved branch (99%) with a cluster containing *C. rudolphii s.l.*, with *C. rudolphii* B basal to all the rest of the sibling species of the complex (Fig. 3).

**Figure 4.**
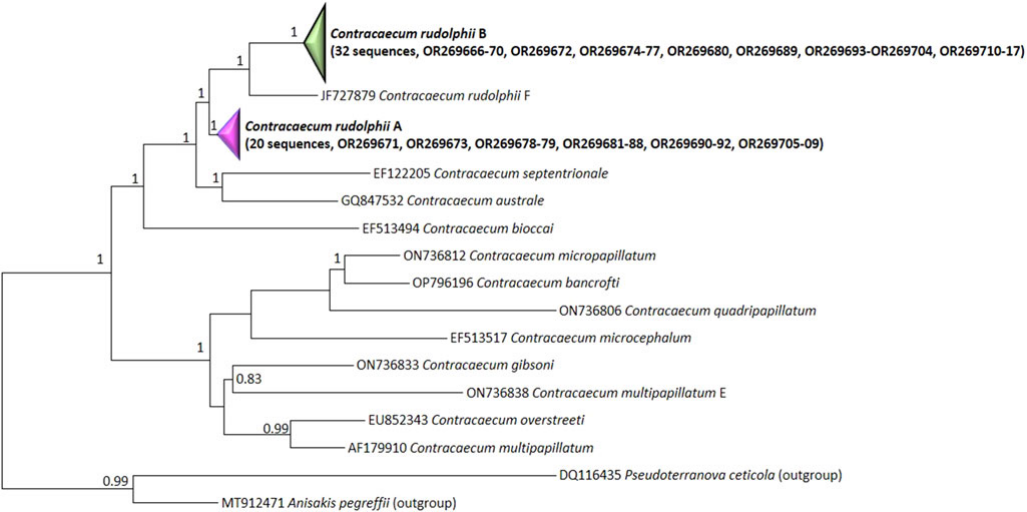
Bayesian inference (BI) tree based on the *cox*2 mtDNA sequences showing the relationship between *C. rudolphii* A (condensed, containing 20 sequences, GB acc. n. OR269671, OR269673, OR269678-79, OR269681-88, OR269690-92, OR269705-09, plus 1 reference sequence MK496476 of *C. rudolphii* A, Mattiucci *et al*., 2020), *C. rudolphii* B (condensed, containing 32 sequences, GenBank accession numbers: OR269666-70, OR269672, OR269674-77, OR269680, OR269689, OR269693-OR269704, OR269710-17, plus 1 reference sequence MK496482 of *C. rudolphii* B, Mattiucci *et al*., 2020) described in the present study (in bold) and the congeneric *Contracaecum* species. The posterior probability is reported for each branch.

Concerning the *cox*2 mtDNA gene, 52 sequences were newly generated (20 *C. rudolphii* A and 32 *C. rudolphii* B). Among *C. rudolphii* A the p-distance was 0–0.2%, while among *C. rudolphii* B was 0–0.4%; between the 2 siblings were 0.8–0.9% as with *C. rudolphii* F (the only *cox*2 available). Moreover, the alignment of the 2 siblings showed the presence of several transitions: 4 A/G, 11 G/A, 1 C/T and 1 T/C (*C. rudolphii* A / *C. rudolphii* B), that are the only variations observed between the 2 siblings. The BLAST search gave 99–100% similarity with the 2 siblings, respectively. The BI inference phylogenetic tree obtained indicate that *C. rudolphii* A and *C. rudolphii* B form 2 distinct clades with high probability values (Fig. 4) including the same siblings retrieved from GB (MK496476 and MK496482, respectively) and with *C. rudolphii* F more related to *C. rudolphii* B. Similarly, the other *Contracaecum* species form a well-supported clade separated from the abovementioned siblings.

**Figure 5.**
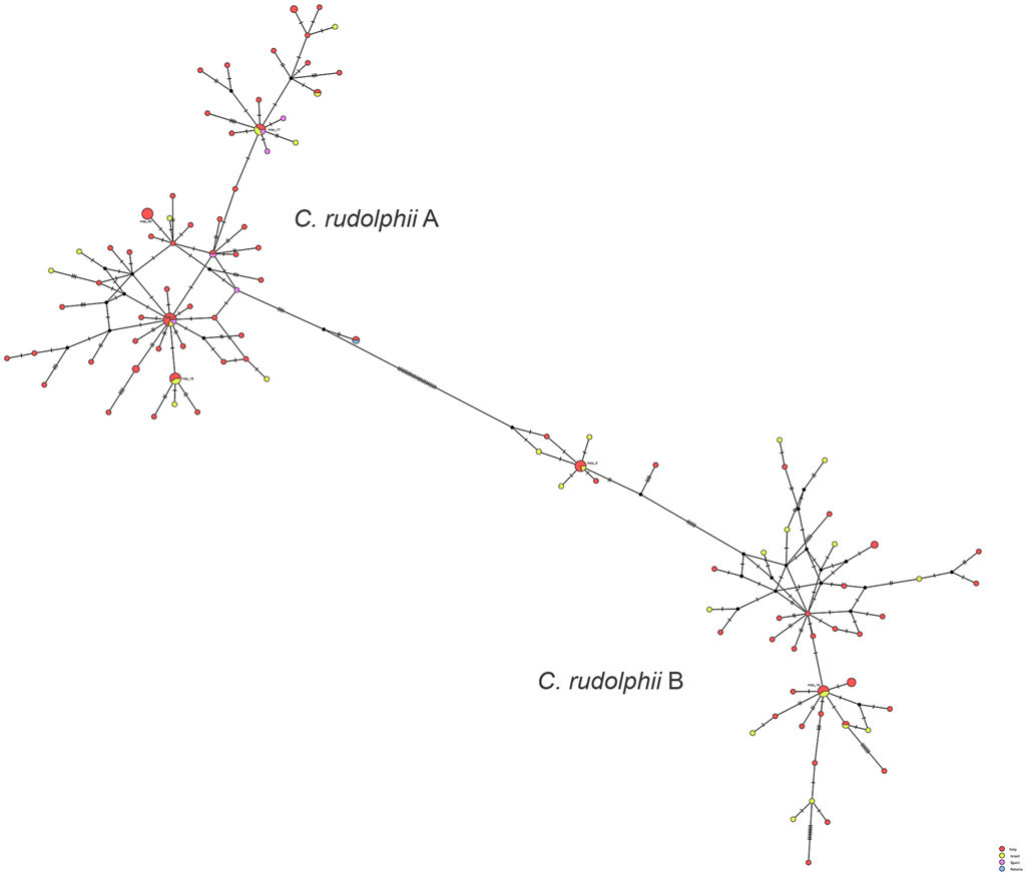
TCS network of haplotypes recorded in the present study, with indications of only the haplotypes detected at least 5 times. All the rest of haplotypes are reported in the supplementary figure S1 and table S1.

Regarding the genetic diversity between the 2 siblings, the number of haplotypes obtained among 141 sequences (83 *C. rudolphii* A + 58 *C. rudolphii* B) from Italy, Israel and Spain (plus 1 from Poland) was 106, of which 92 were private haplotypes with a haplotype's diversity of 0.99 (±0.003) and a nucleotide diversity of 0.044 (±0.0013). Concerning the haplotypes frequency, the most represented is the Hap12 of the *C. rudolphii* A haplogroup shared by 7 specimens from all the geographical regions (Italy, Israel and Spain). The second most representative group of haplotypes are Hap17-18 and 80, all belonging to *C. rudolphii* A haplogroup, containing 5 mixed geographical haplotypes each except the latter (Hap80) composed only by specimens from Sardinia. Finally, the haplotypes 3 and 14 contain *C. rudolphii* B from Italy and Israel. In any case the 2 siblings (A and B) never mixed together. Analysing the genetic diversity among each sibling, we observed in *C. rudolphii* A 60 haplotypes out of 83 sequences analysed, most of them private, with a haplotype's diversity of 0.984 (±0.006) and a nucleotide diversity of 0.0105 (±0.00062); while among *C. rudolphii* B haplotypes were 46 out of 58 sequences with a similar haplotype's diversity of 0.985 ± 0.008 and a nucleotide diversity of 0.0170 (±0.0014). The distribution of the haplotypes frequencies between the 2 siblings is reported in Fig. 5 (see also supplementary material, S1 and S2) as a complex web of haplotypes composed by a double system of star-like network: 1 star encloses the haplogroup A while the other contain the haplogroup B, separated by 26 mutation. No clear geographical differentiation in the distribution of the haplogroups has been detected.

### Morphological descriptions

Morphological analysis allowed to identify all adult males (*n* = 93) and females (*n* = 13) collected as *C. rudolphii s.l*. Morphometric features of specimens identified as *C. rudolphii* A and *C. rudolphii* B by PCR-RFLP and sequencing are reported in Table 2.

**Table 2.** Morphometric features of mature male and female specimens of *C. rudolphii* A and *C. rudolphii* B from *Ph. carbo sinensis* from present study

	Males		Females	
	*C. rudolphii* A	*C. rudolphii* B	*C. rudolphii* A	*C. rudolphii* B
TL	13–28 (22.3 ± 3.5) mm	11–32 (20.6 ± 5.1) mm	26–39 (30.8 ± 4.9)	16–34 (24.7 ± 6.4)
Oe	1906–4190.7 (3451.2 ± 529.6)	1614.9–6051.5 (3299.1 ± 660.3)	3281.4–4188.3 (3910.3 ± 371.3)	2091.9–4360.7 (3200.5 ± 895.2)
IC	1222.1–3374.7 (2551.6 ± 473.3)	1130–4731 (2436.3 ± 525)	2700–3471.5 (3061.2 ± 290.4)	1530.8–3545.4 (2601.5 ± 875.5)
VA	644–1443.1 (1070.4 ± 205.8)	511.4–1456.7 (931.2 ± 187.2)	1096.9–1506.4 (1229.9 ± 182)	667.5–1326 (1011.1 ± 239.7)
NR	298.4–786.6 (593.7 ± 118.6)	274.9–743.2 (534.1 ± 100.9)	502.2–604.4 (563.6 ± 39.2)	401–618.1 (504.4 ± 86.7)
Ta	163.9–365.9 (240.9 ± 42.1)	160.1–336.4 (239 ± 34.4)	346.5–416.4 (377.1 ± 25.7)	303.5–398.7 (345.4 ± 29.3)
RSp	5429.3– 10 217.5 (8054.5 ± 1169.2)	2876.6– 10 580.7 (7527.3 ± 1780.9)	-	-
LSp	5128.2–9985.9 (7755.9 ± 1206.6)	3316.8– 10 091.5 (7449.9 ± 1687.2)	-	-
Vu	-	-	9260.3– 12 807.7 (11 469.3 ± 1456.1)	7243.7– 11 133.5 (8914.2 ± 1346.3)

Measurements are in *μ*m unless otherwise stated.

Abbreviations TL, total length; Oe, oesophagus length; IC, intestinal caecum length; VA, ventricular appendix length; NR, distance of nerve ring from anterior end; Ta, tail length; RSp, right spicule length; LRp, left spicule length; Vu distance of vulva from anterior end.

Main morphological details of *C. rudolphii* s.l. are as follows.

**Figure 6.**
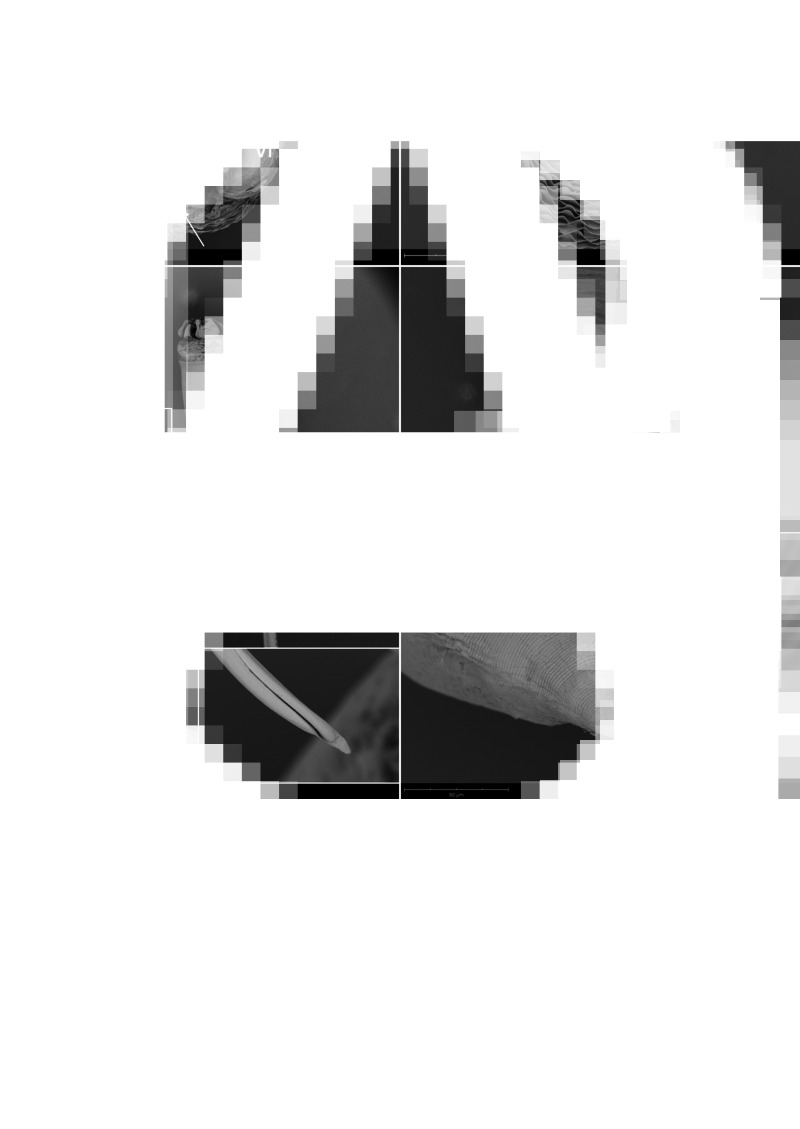
SEM micrographs of *C. rudolphii* A adult male. (A) Apical view of anterior end showing the dorsal lip (dl) and 2 ventral lips (vl) separate by interlabia, and the excretory pore opening at the base of ventral interlabium (arrow). (B) Lateral view of anterior end. (C) Anterior end with detail of the amphid. (D) Caudal end with everted spicules. (E) Spicules with detail of the spicule tip. (F) Caudal end showing the pattern of post-cloacal papillae.

**Figure 7.**
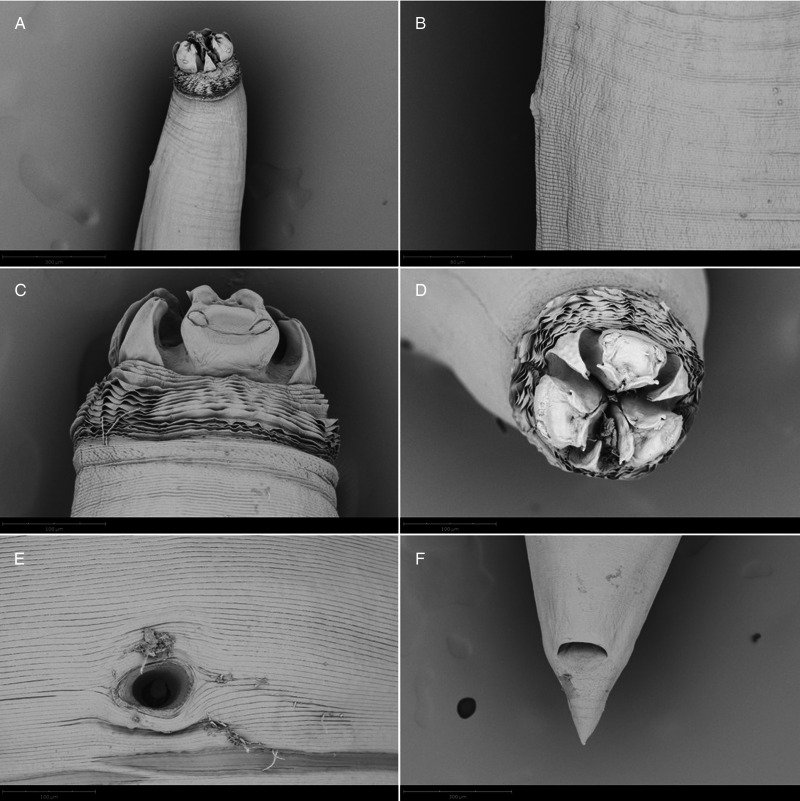
SEM micrographs of *C. rudolphii* B adult female. (A) Ventral view of anterior end. (B) Detail of amphid. (C) Dorsal view of anterior end. (D) Apical view of anterior end. (E) Detail of vulva. (F) Caudal end.

**Figure 8.**
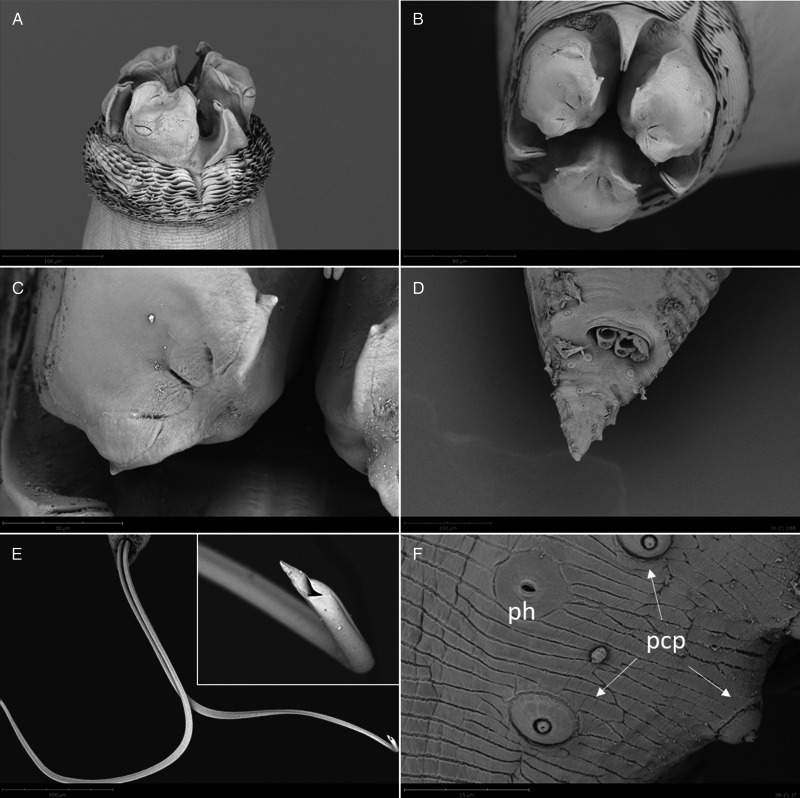
SEM micrographs of *Contracaecum rudolphii* B, adult male. (A) Lateral view of anterior end. (B) Subapical view of anterior end, showing interlabia with bilobed tip. (C) Detail of lip edge. (D) Caudal end showing post cloacal papillae and section of spicules. (E) Caudal end with everted spicules and detail of the spicule tip. (F) Detail of caudal end showing post cloacal papillae (pcp) and phasmid (ph).

**Figure 9.**
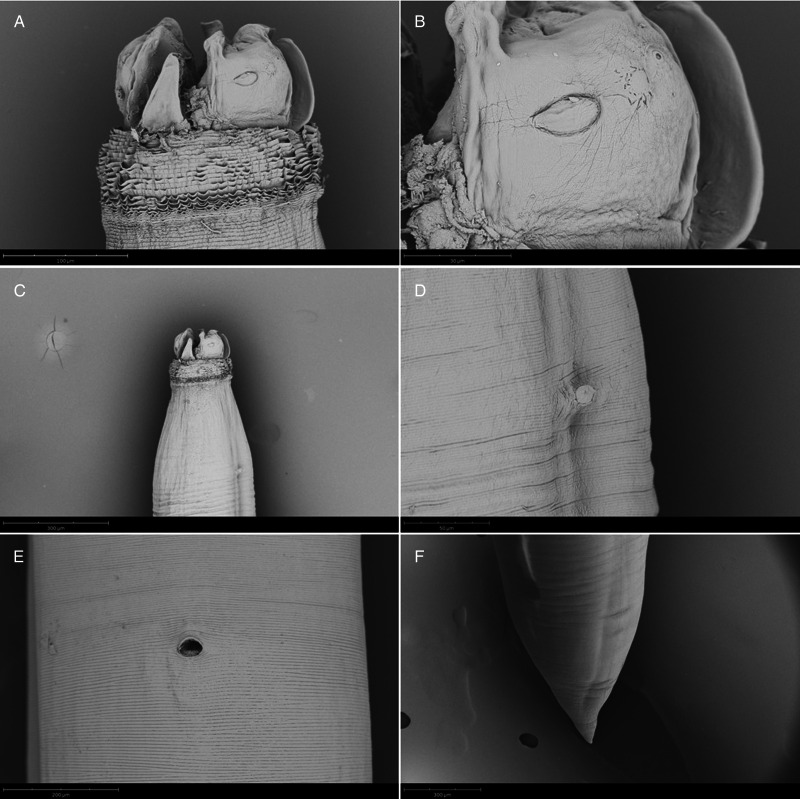
SEM micrographs of *Contracaecum rudolphii* B, adult female. (A) Lateral view of anterior end. (B) Detail of ventral lip. (C) Lateral view of anterior portion. (D) Detail of amphid. (E) Detail of vulva. (F) Caudal end.

Adults with transversely striated cuticle, more marked at anterior end, forming a conspicuous cephalic collar (Figs 6A,B, 7C,D, 8A,B, 9A); 3 well developed lips; 2 pyriform cephalic papillae on dorsal lip (Figs 6A, 7C), 1 cephalic papilla on each subventral lip (Figs 6B, 8A, 9A,B); interlabia well developed, with large base and bilobed tip (Fig. 8B); excretory pore opening at base of ventral interlabium (Fig. 6A); oesophagus muscular, with small globular ventricle; ventricular appendix directed posteriorly; intestinal caecum 2 to 3 times longer than ventricular appendix, directed anteriorly.

Males with tail conical, curved at tip (Figs 6F, 8D), with 27–40 pairs of pre-cloacal papillae, forming 2 subventral lines, 2 pairs of paracloacal papillae, 2 pairs of distal subventral papillae and 2 pairs of distal sublateral papillae, 1 pair of phasmids (Figs 6F, 8D, F); spicules sub-equal, folded, with longitudinal alae and pointed tips (Figs 6D,E, 8D,E).

Females larger than males; vulva around second quarter of body length (Figs 7E, 9E); tail conical (Figs 7F, 9F), with rounded tip. Eggs subspherical.

## Discussion

The lack of morphological features useful to discriminate among the *C. rudolphii* A and *C. rudolphii* B confirms the need of coupling the traditional parasitological observations with the molecular approach based at least on 2 molecular markers, e.g. ITS rDNA and *cox*2 mtDNA to reach the correct identification.

All adults collected from the *Ph. carbo sinensis* sampled in Italy and Israel belong to *C. rudolphii s.l*. confirming great cormorant as the main definitive host for this complex of species in EU (Amor *et al*., 2020; Mattiucci *et al*., 2020; Carmeno *et al*., 2022; Cammilleri *et al*., 2023) as well as in Israel, where *C. rudolphii s.l*. had never been described.

The molecular analyses (PCR-RFLP and sequencing) performed on 683 adults from 28 *Ph. carbo sinensis* (10 from Italy and 18 from Israel) allowed the identification of the sibling species *C. rudolphii* A and *C. rudolphii* B, often in mixed infections (Table 1). The co-occurrence of both species in the same bird host has been already reported in other studies (Szostakowska *et al*., 2002; Szostakowska and Fagerholm, 2007; Amor *et al*., 2020; Mattiucci *et al*., 2020; Carmeno *et al*., 2022; Cammilleri *et al*., 2023); similar distributions have been observed also in our study. In Italian specimens, the 2 siblings showed a similar proportion (184 A *vs* 195 B), while the birds from Israel were more frequently infected with *C. rudolphii* B (116 A *vs* 188 B) with only 4 birds out of 18 examined showing a mixed infection.

The phylogenetical analyses of both genetic markers showed a well-supported separation between *C. rudolphii* A and *C. rudolphii* B, with *C. rudolphii* B more closely related to *C. rudolphii* F than to *C. rudolphii* A in the *cox*2 (Amor *et al*., 2020; Mattiucci *et al*., 2020; Roca-Geronès *et al*., 2023), but not in ITS rDNA. Unfortunately, except for *C. rudolphii* F, no *cox*2 mtDNA sequences are available for the other siblings (C, D, E) included in *C. rudolphii* complex to better clarify the relationship among the complex even by this more evolving gene.

Concerning the population structure, we observed a high genetic variability as demonstrated by the high numbers of haplotypes in both *C. rudolphii* A (83 sequences/60 haplotypes) and *C. rudolphii* B (58 sequences/46 haplotypes) populations; similar results were obtained also by Amor *et al*. (2020) in Sardinia (Italy) for *cox*2 mtDNA (*C. rudolphii* A: n. haplotypes 33 out of 158, hd = 0.985, nucleotide diversity = 0.013; *C. rudolphii* B: nucleotide diversity 7/22, hd = 0.952, nucleotide diversity = 0.021). In Spain, Roca-Geronès *et al*. (2023) analysed only *C. rudolphii* A, that was considered as single population due to the low genetic diversity (n. haplotypes 40/56, hd = 0.969, nucleotide diversity = 0.00681). In the same study, the comparisons with the sequences of *C. rudolphii* A from 2 Italian areas showed similar values as obtained in the present study (Tyrrhenian: n. haplotypes 80/110, hd = 0.986, nucleotide diversity = 0.00871; western Sardinia: n. haplotypes 28/33, hd = 0.979, nucleotide diversity = 0.0117). The haplotypes diversity in any case reflect the geographical distributions of the sequences analysed (Amor *et al*., 2020; Roca-Geronès *et al*., 2023). Amor *et al*. (2020) speculate about the low genetic diversity among the 2 siblings, suggesting that the high infection rates in definitive hosts slow down the genetic drift, but also it could be due to the parasites dispersal model mediated by the host dispersal dynamic. The migrations of the definitive hosts and the population structure could influence the genetic structure of this Anisakidae, as reported by Cipriani *et al*. (2022) for *Anisakis* spp.

The SEM observations allowed for the first time a detailed characterization of the external features of *C. rudolphii* B, and added further morphological information about *C. rudolphii* A, which was already examined using SEM by Abollo *et al*. (2001). Particularly, we provide the first detailed SEM characterization of the spicule morphology in both sibling species, with special reference to their folded appearance and to the morphology of the distal extremity.

Overall, compared to other siblings of the species complex (Abollo *et al*., 2001; Shamsi *et al*., 2009) and to *C. rudolphii s.l.* (Amato *et al*., 2006; Li *et al*., 2013) previously analysed by SEM, our specimens show a similar external morphology of anterior and posterior extremity. The pattern of post-cloacal papillae in adult males and the conspicuous cuticular collar in both males and females are particularly distinctive characters that support the identification of *C. rudolphii s.l.*

Generally, cormorants from central Italy showed a similar proportion of adult stages of *C. rudolphii* A and *C. rudolphii* B while the ones from Israel showed a higher proportion of *C. rudolphii* B. It has been suggested that the different feeding ecology and wintering behaviour of different populations of *Ph. carbo sinensis*, could be one of the ‘drivers’ of the differential spatial distribution of *C. rudolphii* A and *C. rudolphii* B in the different aquatic ecosystems, i.e. brackish/marine and freshwater environments, respectively (Mattiucci *et al*., 2020). Moreover, abiotic factors related to early stages of the parasites, have been supposed to contribute to the differential occurrence of the 2 sibling species in the 2 aquatic ecosystems (Moravec, 2009; Mattiucci *et al*., 2020).

Recent work suggested the possible role of the migration routes of wintering populations of cormorants in the Mediterranean Sea in influencing the distribution and genetic structure of *C. rudolphii* (Roca-Geronès *et al*., 2023). Cormorants migrate from Europe and winter in Israel during November–March, forming large colonies along the Mediterranean and Red Sea coasts and at inland streams and wetlands (Nemtzov, 2008). Large numbers of *C. rudolphii* A could be acquired by cormorants feeding in coastal areas during their migration across the Mediterranean Sea. Furthermore, most of Israel's wetlands are exploited for fish farming, mainly in freshwater fishponds; these fish are particularly susceptible to predation by piscivorous birds during migration stopovers (Nemtzov, 2002) and could provide opportunities for maintaining the life cycle of *C. rudolphii* B. In our study, all the great cormorants from both countries have been sampled in winter; interestingly, in 2 cases the birds from Israel were sampled in an area very close to the sea but they were infected only by *C. rudolphii* B, therefore we could hypothesize that they arrived in these wintering sites already parasitized. To date, larval stages of *C. rudolphii* have never been reported in marine/brackish/freshwater fish from Israel. This could either be due to biotic/abiotic factors unfavourable to the development of *C. rudolphii* larvae, or to difficulties in the detection of small larval stages (much smaller than those of other *Contracaecum* spp.) during field inspections. Despite their adaptation to different aquatic ecosystems, larvae of both *C. rudolphii* A and *C. rudolphii* B have generally shown a low specificity for their fish host (Mattiucci *et al*., 2020), therefore the availability of suitable paratenic hosts does not seem to be a limiting factor for the successful establishment of the parasite life cycle.

This work presents the first data on the occurrence of sibling species of *C. rudolphii* in Israel and provides additional data on the distribution of *C. rudolphii* A and B in Italy, confirming the high prevalence and intensity of infection observed in *Ph. carbo sinensis* from other Italian areas. Based on these results, we stress the importance of carrying out targeted investigations aimed at evaluating the occurrence of *C. rudolphii* in marine and freshwater fish from Israel, to understand the epidemiology of the parasite in this important wintering stopover.

## Supporting information

Caffara et al. supplementary material 1 Caffara et al. supplementary material

Caffara et al. supplementary material 2 Caffara et al. supplementary material

## Data Availability

The DNA sequences generated in this study have been deposited on the public database GenBank under accession numbers OR263194-OR263246 (ITS rDNA) and OR269666-OR269717 (*cox*2 mtDNA).

## Supplementary material

The supplementary material for this article can be found at https://doi.org/10.1017/S0031182023000902
