# Supplementary material for: Molecular and morphological studies on Contracaecum rudolphii A and C. rudolphii B in great cormorants (Phalacrocorax carbo sinensis) from Italy and Israel
Source: Parasitology. 2023 Sep 21;150(11):1040–51. doi: 10.1017/S0031182023000902 (PMC10941213; doi:10.1017/S0031182023000902)
Supplement: Caffara et al. supplementary material 1 — Caffara et al. supplementary material [file S0031182023000902sup001.pdf]

GB acc n. cox2	code_haplo	n. haplotypes	Hosts species	Locality	Contracaecum rudolphii sibling	References
OR269668	ITB33	Hap_3	*Ph. carbo sinensis*	Italy	C. rudolphii B	present study
OR269700	ISB44	Hap_3	*Ph. carbo sinensis*	Israel	C. rudolphii B	present study
EF558893	ITB66	Hap_3	*Ph. carbo sinensis*	Italy	C. rudolphii B	Mattiucci et al., 2008
EF122203	ITB68	Hap_3	*Ph. carbo sinensis*	Italy	C. rudolphii B	Mattiucci et al., 2008
MK496485	ITB94	Hap_3	*Ph. carbo sinensis*	Italy	C. rudolphii B	Mattiucci et al., 2020
OR269669	ITB30	Hap_4	*Ph. carbo sinensis*	Italy	C. rudolphii B	present study
OR269672	ITB52	Hap_4	*Ph. carbo sinensis*	Italy	C. rudolphii B	present study
EF513506	ITB74	Hap_4	*Ph. carbo sinensis*	Italy	C. rudolphii B	Mattiucci et al., 2008
OR269673	ITA49	Hap_7	*Ph. carbo sinensis*	Italy	C. rudolphii A	present study
MT484280	ITA149	Hap_7	*Sparus aurata*	Italy	C. rudolphii A	Guardone et al., 2020
OR269677	ISB4	Hap_11	*Ph. carbo sinensis*	Israel	C. rudolphii B	present study
MK496484	ITB95	Hap_11	*Ph. carbo sinensis*	Italy	C. rudolphii B	Mattiucci et al., 2020
OR269678	ISA5	Hap_12	*Ph. carbo sinensis*	Israel	C. rudolphii A	present study
OR269706	ITA26	Hap_12	*Ph. carbo sinensis*	Italy	C. rudolphii A	present study
OP690537	SPA89	Hap_12	*Ph. aristotelis desmarestiis*	Spain	C. rudolphii A	Roca-Geronès et al., 2023
MK496478	ITA101	Hap_12	*Ph. carbo sinensis*	Italy	C. rudolphii A	Mattiucci et al., 2020
MT339006	ITA111	Hap_12	*Ph. carbo sinensis*	Italy	C. rudolphii A	Amor et al., 2020
MT339008	ITA113	Hap_12	*Ph. carbo sinensis*	Italy	C. rudolphii A	Amor et al., 2020
MT339014	ITA119	Hap_12	*Ph. carbo sinensis*	Italy	C. rudolphii A	Amor et al., 2020
OR269680	ISB7	Hap_14	*Ph. carbo sinensis*	Israel	C. rudolphii B	present study
OR269699	ISB43	Hap_14	*Ph. carbo sinensis*	Israel	C. rudolphii B	present study
EF558895	ITB64	Hap_14	*Ph. carbo sinensis*	Italy	C. rudolphii B	Mattiucci et al., 2008
EF558894	ITB65	Hap_14	*Ph. carbo sinensis*	Italy	C. rudolphii B	Mattiucci et al., 2008
EF513508	ITB72	Hap_14	*Ph. carbo sinensis*	Italy	C. rudolphii B	Mattiucci et al., 2008
OR269682	ISA10	Hap_16	*Ph. carbo sinensis*	Israel	C. rudolphii A	present study
MT484279	ITA148	Hap_16	*Sparus aurata*	Italy	C. rudolphii A	Guardone et al., 2020
OR269683	ISA8	Hap_17	*Ph. carbo sinensis*	Israel	C. rudolphii A	present study
OR269692	ISA36	Hap_17	*Ph. carbo sinensis*	Israel	C. rudolphii A	present study
OP690538	SPA88	Hap_17	*Ph. aristotelis desmarestiis*	Spain	C. rudolphii A	Roca-Geronès et al., 2023
MK496480	ITA99	Hap_17	*Ph. carbo sinensis*	Italy	C. rudolphii A	Mattiucci et al., 2020
MT339009	ITA114	Hap_17	*Ph. carbo sinensis*	Italy	C. rudolphii A	Amor et al., 2020

OR269684	ISA11	Hap_18	*Ph. carbo sinensis*	Israel	C. rudolphii A	present study
OR269686	ISA13	Hap_18	*Ph. carbo sinensis*	Israel	C. rudolphii A	present study
EF513504	ITA76	Hap_18	*Ph. carbo sinensis*	Italy	C. rudolphii A	Mattiucci et al., 2008
EF558891	ITA83	Hap_18	*Ph. carbo sinensis*	Italy	C. rudolphii A	Mattiucci et al., 2008
MT339027	ITA132	Hap_18	*Ph. carbo sinensis*	Italy	C. rudolphii A	Amor et al., 2020
OR269709	ITA29	Hap_38	*Ph. carbo sinensis*	Italy	C. rudolphii A	present study
MT339024	ITA129	Hap_38	*Ph. carbo sinensis*	Italy	C. rudolphii A	Amor et al., 2020
EF558896	ITB63	Hap_47	*Ph. carbo sinensis*	Italy	C. rudolphii B	Mattiucci et al., 2008
EF513511	ITB69	Hap_47	*Ph. carbo sinensis*	Italy	C. rudolphii B	Mattiucci et al., 2008
EF558892	POA82	Hap_58	*Ph. carbo sinensis*	Poland	C. rudolphii A	Mattiucci et al., 2008
EF122201	ITA85	Hap_58	*Ph. carbo sinensis*	Italy	C. rudolphii A	Mattiucci et al., 2008
OP690540	SPA86	Hap_68	*Ph. aristotelis desmarestiis*	Spain	C. rudolphii A	Roca-Geronès et al., 2023
MT339001	ITA106	Hap_68	*Ph. carbo sinensis*	Italy	C. rudolphii A	Amor et al., 2020
MT339011	ITA116	Hap_80	*Ph. carbo sinensis*	Italy	C. rudolphii A	Amor et al., 2020
MT339016	ITA121	Hap_80	*Ph. carbo sinensis*	Italy	C. rudolphii A	Amor et al., 2020
MT339017	ITA122	Hap_80	*Ph. carbo sinensis*	Italy	C. rudolphii A	Amor et al., 2020
MT339018	ITA123	Hap_80	*Ph. carbo sinensis*	Italy	C. rudolphii A	Amor et al., 2020
MT339019	ITA124	Hap_80	*Ph. carbo sinensis*	Italy	C. rudolphii A	Amor et al., 2020
OR269676	ISB1	Hap_10	*Ph. carbo sinensis*	Israel	C. rudolphii B	present study
OR269674	ISB2	Hap_8	*Ph. carbo sinensis*	Israel	C. rudolphii B	present study
OR269675	ISB3	Hap_9	*Ph. carbo sinensis*	Israel	C. rudolphii B	present study
OR269679	ISA6	Hap_13	*Ph. carbo sinensis*	Israel	C. rudolphii A	present study
OR269681	ISA9	Hap_15	*Ph. carbo sinensis*	Israel	C. rudolphii A	present study
OR269685	ISA12	Hap_19	*Ph. carbo sinensis*	Israel	C. rudolphii A	present study
OR269687	ISA14	Hap_20	*Ph. carbo sinensis*	Israel	C. rudolphii A	present study
OR269688	ISA15	Hap_21	*Ph. carbo sinensis*	Israel	C. rudolphii A	present study
OR269689	ISB16	Hap_22	*Ph. carbo sinensis*	Israel	C. rudolphii B	present study
OR269690	ISA17	Hap_23	*Ph. carbo sinensis*	Israel	C. rudolphii A	present study
OR269710	ITB18	Hap_39	*Ph. carbo sinensis*	Italy	C. rudolphii B	present study
OR269711	ITB19	Hap_40	*Ph. carbo sinensis*	Italy	C. rudolphii B	present study
OR269712	ITB20	Hap_41	*Ph. carbo sinensis*	Italy	C. rudolphii B	present study
OR269713	ITB21	Hap_42	*Ph. carbo sinensis*	Italy	C. rudolphii B	present study
OR269714	ITB22	Hap_43	*Ph. carbo sinensis*	Italy	C. rudolphii B	present study

OR269715	ITB23	Hap_44	*Ph. carbo sinensis*	Italy	C. rudolphii B	present study
OR269716	ITB24	Hap_45	*Ph. carbo sinensis*	Italy	C. rudolphii B	present study
OR269705	ITA25	Hap_35	*Ph. carbo sinensis*	Italy	C. rudolphii A	present study
OR269707	ITA27	Hap_36	*Ph. carbo sinensis*	Italy	C. rudolphii A	present study
OR269708	ITA28	Hap_37	*Ph. carbo sinensis*	Italy	C. rudolphii A	present study
OR269717	ITB31	Hap_46	*Ph. carbo sinensis*	Italy	C. rudolphii B	present study
OR269666	ITB32	Hap_1	*Ph. carbo sinensis*	Italy	C. rudolphii B	present study
OR269667	ITB34	Hap_2	*Ph. carbo sinensis*	Italy	C. rudolphii B	present study
OR269691	ISA35	Hap_24	*Ph. carbo sinensis*	Israel	C. rudolphii A	present study
OR269694	ISB37	Hap_26	*Ph. carbo sinensis*	Israel	C. rudolphii B	present study
OR269693	ISB38	Hap_25	*Ph. carbo sinensis*	Israel	C. rudolphii B	present study
OR269695	ISB39	Hap_27	*Ph. carbo sinensis*	Israel	C. rudolphii B	present study
OR269696	ISB40	Hap_28	*Ph. carbo sinensis*	Israel	C. rudolphii B	present study
OR269697	ISB41	Hap_29	*Ph. carbo sinensis*	Israel	C. rudolphii B	present study
OR269698	ISB42	Hap_30	*Ph. carbo sinensis*	Israel	C. rudolphii B	present study
OR269702	ISB45	Hap_32	*Ph. carbo sinensis*	Israel	C. rudolphii B	present study
OR269701	ISB46	Hap_31	*Ph. carbo sinensis*	Israel	C. rudolphii B	present study
OR269703	ISB47	Hap_33	*Ph. carbo sinensis*	Israel	C. rudolphii B	present study
OR269704	ISB48	Hap_34	*Ph. carbo sinensis*	Israel	C. rudolphii B	present study
OR269670	ITB50	Hap_5	*Ph. carbo sinensis*	Italy	C. rudolphii B	present study
OR269671	ITA51	Hap_6	*Ph. carbo sinensis*	Italy	C. rudolphii A	present study
EF122204	ITB67	Hap_48	*Ph. carbo sinensis*	Italy	C. rudolphii B	Mattiucci et al., 2008
EF513510	ITB70	Hap_49	*Ph. carbo sinensis*	Italy	C. rudolphii B	Mattiucci et al., 2008
EF513509	ITB71	Hap_50	*Ph. carbo sinensis*	Italy	C. rudolphii B	Mattiucci et al., 2008
EF513507	ITB73	Hap_51	*Ph. carbo sinensis*	Italy	C. rudolphii B	Mattiucci et al., 2008
EF513505	ITA75	Hap_52	*Ph. carbo sinensis*	Italy	C. rudolphii A	Mattiucci et al., 2008
EF513503	ITA77	Hap_53	*Ph. carbo sinensis*	Italy	C. rudolphii A	Mattiucci et al., 2008
EF513502	ITA78	Hap_54	*Ph. carbo sinensis*	Italy	C. rudolphii A	Mattiucci et al., 2008
EF513501	ITA79	Hap_55	*Ph. carbo sinensis*	Italy	C. rudolphii A	Mattiucci et al., 2008
EU852349	ITB80	Hap_56	*Ph. carbo sinensis*	Italy	C. rudolphii B	Mattiucci et al., 2008
EF535570	ITA81	Hap_57	*Ph. carbo sinensis*	Italy	C. rudolphii A	Mattiucci et al., 2008
EF122202	ITA84	Hap_59	*Ph. carbo sinensis*	Italy	C. rudolphii A	Mattiucci et al., 2008
OP690539	SPA87	Hap_69	*Ph. aristotelis desmarestiis*	Spain	C. rudolphii A	Roca-Geronès et al., 2023

OP690536	SPA90	Hap_70	*Ph. aristotelis desmarestiis*	Spain	C. rudolphii A	Roca-Geronès et al., 2023
OP690535	SPA91	Hap_71	*Ph. aristotelis desmarestiis*	Spain	C. rudolphii A	Roca-Geronès et al., 2023
MK496487	ITB92	Hap_60	*Ph. carbo sinensis*	Italy	C. rudolphii B	Mattiucci et al., 2020
MK496486	ITB93	Hap_61	*Ph. carbo sinensis*	Italy	C. rudolphii B	Mattiucci et al., 2020
MK496483	ITB96	Hap_62	*Ph. carbo sinensis*	Italy	C. rudolphii B	Mattiucci et al., 2020
MK496482	ITB97	Hap_63	*Ph. carbo sinensis*	Italy	C. rudolphii B	Mattiucci et al., 2020
MK496481	ITA98	Hap_64	*Ph. carbo sinensis*	Italy	C. rudolphii A	Mattiucci et al., 2020
MK496479	ITA100	Hap_65	*Ph. carbo sinensis*	Italy	C. rudolphii A	Mattiucci et al., 2020
MK496477	ITA102	Hap_66	*Ph. carbo sinensis*	Italy	C. rudolphii A	Mattiucci et al., 2020
MK496476	ITA103	Hap_67	*Ph. carbo sinensis*	Italy	C. rudolphii A	Mattiucci et al., 2020
MT338999	ITA104	Hap_72	*Ph. carbo sinensis*	Italy	C. rudolphii A	Amor et al., 2020
MT339000	ITA105	Hap_73	*Ph. carbo sinensis*	Italy	C. rudolphii A	Amor et al., 2020
MT339002	ITA107	Hap_74	*Ph. carbo sinensis*	Italy	C. rudolphii A	Amor et al., 2020
MT339003	ITA108	Hap_75	*Ph. carbo sinensis*	Italy	C. rudolphii A	Amor et al., 2020
MT339004	ITA109	Hap_76	*Ph. carbo sinensis*	Italy	C. rudolphii A	Amor et al., 2020
MT339005	ITA110	Hap_77	*Ph. carbo sinensis*	Italy	C. rudolphii A	Amor et al., 2020
MT339007	ITA112	Hap_78	*Ph. carbo sinensis*	Italy	C. rudolphii A	Amor et al., 2020
MT339010	ITA115	Hap_79	*Ph. carbo sinensis*	Italy	C. rudolphii A	Amor et al., 2020
MT339012	ITA117	Hap_81	*Ph. carbo sinensis*	Italy	C. rudolphii A	Amor et al., 2020
MT339013	ITA118	Hap_82	*Ph. carbo sinensis*	Italy	C. rudolphii A	Amor et al., 2020
MT339015	ITA120	Hap_83	*Ph. carbo sinensis*	Italy	C. rudolphii A	Amor et al., 2020
MT339020	ITA125	Hap_84	*Ph. carbo sinensis*	Italy	C. rudolphii A	Amor et al., 2020
MT339021	ITA126	Hap_85	*Ph. carbo sinensis*	Italy	C. rudolphii A	Amor et al., 2020
MT339022	ITA127	Hap_86	*Ph. carbo sinensis*	Italy	C. rudolphii A	Amor et al., 2020
MT339023	ITA128	Hap_87	*Ph. carbo sinensis*	Italy	C. rudolphii A	Amor et al., 2020
MT339025	ITA130	Hap_88	*Ph. carbo sinensis*	Italy	C. rudolphii A	Amor et al., 2020
MT339026	ITA131	Hap_89	*Ph. carbo sinensis*	Italy	C. rudolphii A	Amor et al., 2020
MT339028	ITA133	Hap_90	*Ph. carbo sinensis*	Italy	C. rudolphii A	Amor et al., 2020
MT339029	ITA134	Hap_91	*Ph. carbo sinensis*	Italy	C. rudolphii A	Amor et al., 2020
MT339030	ITA135	Hap_92	*Ph. carbo sinensis*	Italy	C. rudolphii A	Amor et al., 2020
MT339031	ITA136	Hap_93	*Ph. carbo sinensis*	Italy	C. rudolphii A	Amor et al., 2020
MT339032	ITB137	Hap_94	*Ph. carbo sinensis*	Italy	C. rudolphii B	Amor et al., 2020
MT339033	ITB138	Hap_95	*Ph. carbo sinensis*	Italy	C. rudolphii B	Amor et al., 2020

MT339034	ITB139	Hap_96	*Ph. carbo sinensis*	Italy	C. rudolphii B	Amor et al., 2020
MT339035	ITB140	Hap_97	*Ph. carbo sinensis*	Italy	C. rudolphii B	Amor et al., 2020
MT339036	ITB141	Hap_98	*Ph. carbo sinensis*	Italy	C. rudolphii B	Amor et al., 2020
MT339037	ITB142	Hap_99	*Ph. carbo sinensis*	Italy	C. rudolphii B	Amor et al., 2020
MT339038	ITB143	Hap_100	*Ph. carbo sinensis*	Italy	C. rudolphii B	Amor et al., 2020
MT484275	ITA144	Hap_101	*Dicentrarchus labrax*	Italy	C. rudolphii A	Guardone et al., 2020
MT484276	ITA145	Hap_102	*Dicentrarchus labrax*	Italy	C. rudolphii A	Guardone et al., 2020
MT484277	ITA146	Hap_103	*Dicentrarchus labrax*	Italy	C. rudolphii A	Guardone et al., 2020
MT484278	ITA147	Hap_104	*Dicentrarchus labrax*	Italy	C. rudolphii A	Guardone et al., 2020
MT484281	ITA150	Hap_105	*Sparus aurata*	Italy	C. rudolphii A	Guardone et al., 2020
MT484282	ITA151	Hap_106	*Sparus aurata*	Italy	C. rudolphii A	Guardone et al., 2020
