# Supplementary figures and images for: Molecular and morphological studies on Contracaecum rudolphii A and C. rudolphii B in great cormorants (Phalacrocorax carbo sinensis) from Italy and Israel
Source: Parasitology. 2023 Sep 21;150(11):1040–51. doi: 10.1017/S0031182023000902 (PMC10941213; doi:10.1017/S0031182023000902)

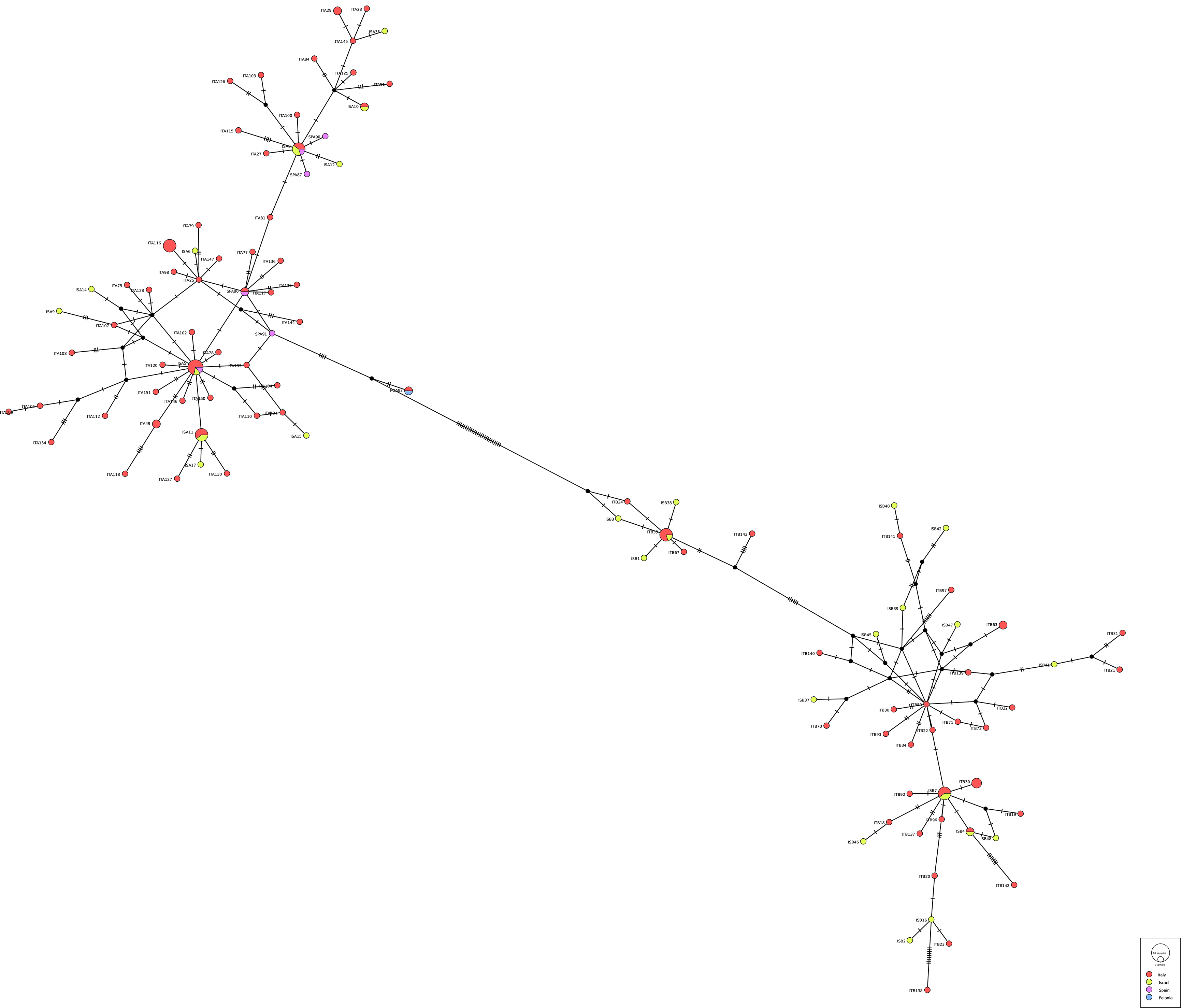

Supplement: Caffara et al. supplementary material 2 — Caffara et al. supplementary material [file S0031182023000902sup002.jpg]
